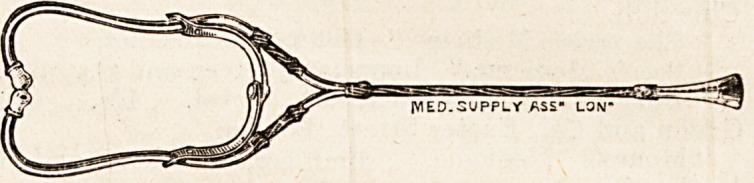# Microscopes

**Published:** 1907-09-07

**Authors:** 


### MICROSCOPES.

A good microscope is an indispensable instrument for the student and practitioner, and the choice of one is not easy in view of the vast number of different makes which compete for admiration, each of which possesses particular claims for attention and consideration. The following are the chief makers, whose instruments without exception are thoroughly reliable and suitable for both students and practitioners :—

E. Leitz; Wetzlar (London offices, 9 Oxford Street). Stands F and C are recommended, the latter being very suitable for research work. Prices from £8 5s. to £25, according to accessories and number of objectives.

W. F. Stanley and Co., 13 Railway Approach, London Bridge, supply a student's model (£5 5s.), which is excellent for all ordinary purposes. The firm also lists more expensive models, and supplies instruments by other makers.

Swift and Son, 81 Tottenham Court Road, W., sell newly designed instruments for histological and physiological work, fitted with their patent "Ariston" fine adjustment. Their £15 bacteriological microscope is a fine instrument, which is thoroughly to be recommended.

Bausch and Lomb Optical Co. (Agents, A. E. Staley, 19 Thavies Inn, Holborn Circus). The instruments supplied by this firm are exceptionally fine. Stands CD, CA, and DD are suitable for advanced work, while Stands BA, BH, and B are recommended for students' use.

W. Watson and Sons, 313 High Holborn, are makers of high-class microscopes, objectives, and eye-pieces. Their "Fram," "Praxis," "Royal," and "Edinburgh" student models, at prices ranging from £8 upwards, are thoroughly reliable instruments.

Voigtländer and Sohn, 12 Charterhouse Street, E.C. This firm lists a particularly good instrument (Stand III.) with objectives and accessories complete at £9 10s., and the instrument is eminently suitable for students' use. More elaborate stands are manufactured by the firm at prices which, considering the excellence of the instruments, are by no means expensive.

Carl Zeiss, Jena, 29 Margaret Street, Regent Street, W. "Zeiss" is a name deservedly honoured among microscopists. The instruments supplied by this firm are second to none in beauty of finish and reliability, and are well worth the slightly higher prices demanded for them. The firm stocks special complete outfits. Stand I. is one of the finest on the market, and Stands IV. and V. are designed for students or those who desire a less elaborate instrument at a lower price.

Messrs. Ross, Limited, 111 New Bond Street, W., are well-known makers, whose instruments are highly spoken of by those who have used them. Their No. 2A outfit, listed at £10 15s., is recommended for students' use, while outfits Nos. 2C, E, and F, at prices ranging from £18 10s. to £22 12s., are excellent instruments for clerical and research work in bacteriology and hæmatology.

Messrs. R. and J. Beck, Limited, 68 Cornhill, London, are makers of the fine "Imperial," "London," and "British Student" stands, all of which are thoroughly to be recommended. Special outfits, which include these stands and all necessary accessories, are listed by the firm at prices ranging from £4 15s. to £72 11s.

### A NEW BINAURAL STETHOSCOPE.

(Medical Supply Association, 228 Gray's Inn Road.)

We have given this instrument a careful trial. As shown in the illustration, it differs from the ordinary type of binaural stethoscope in that it possesses a central "stem" of tubing the bifurcation of which is placed much closer to the ear-pieces than in the ordinary instrument. The chest-piece is large, and is made to unscrew so as to admit of intercostal auscultation, and the distal ends of the metal tubes curve inwards instead of outwards. The instrument is from designs of Mr. J. Inman Langley, M.R.C.S., L.R.C.P., who claims that it is neater in appearance, more convenient, and more compact than the binaural stethoscope ordinarily used. There can be no question that the third claim is well founded. The stethoscope folds up very compactly, and can easily be slipped into a side pocket. In clinical use we have found it an excellent instrument. It does not appear to exaggerate normal sounds, while murmurs of low intensity are well transmitted by it. The chest-piece necessitates the use of two fingers to hold it securely on the surface of the thorax, and, as the instrument lacks the V-shaped lower extremity, which is so useful for steadying the instrument with one finger, it should be modified by the addition of a central finger-piece. The instrument is well and strongly made, and is listed at 10s. 6d.

### MILLIKIN AND LAWLEY.

To most medical practitioners and students Millikin and Lawley, "dealers in surgical instruments, microscopes, and osteology," are well known. The firm issues monthly catalogues of new and second-hand instruments, appliances, microscopes, and bones, and the student cannot do better than give his careful attention to these. The prices quoted are in every case reasonable : the goods supplied, as we have experienced, are in every case reliable and trustworthy. A feature is the selection of microscopes of which Millikin and Lawley stock a large number, both new and second-hand, including stands by all the leading makers. Students' requisites, such as sets and half-sets of bones, dissecting cases, and dressing instruments, are well represented in the catalogue, which is forwarded free on application to the firm's emporium, 165 Strand, W.C.

## Figures and Tables

**Figure f1:**